# Carcinoma-associated fibroblasts release microRNA-331-3p containing extracellular vesicles to exacerbate the development of pancreatic cancer *via* the SCARA5-FAK axis

**DOI:** 10.1080/15384047.2022.2041961

**Published:** 2022-05-05

**Authors:** Yadong Han, Xu Qian, Teng Xu, Yang Shi

**Affiliations:** a Department of General Surgery, The First Affiliated Hospital of Soochow University, Suzhou, Jiangsu Province, China; b Department of General Surgery, The Affiliated Hospital of Xuzhou Medical University, Xuzhou , China

**Keywords:** Carcinoma-associated fibroblasts, extracellular vesicles, miR-331-3p, SCARA5, pancreatic cancer, FAK pathway

## Abstract

microRNA-331-3p (miR-331-3p) has been displayed as an oncogene in pancreatic cancer (PC). The current research set out to elucidate how miR-331-3p in carcinoma-associated fibroblasts (CAFs)-derived extracellular vesicles (EVs) facilitated the tumorigenesis in PC. First, a dual-luciferase reporter assay was adopted to investigate the relationship between miR-331-3p and SCARA5. In addition, EVs were isolated normal fibroblasts and CAFs, and these isolated EVs were co-cultured with PC cells. Cell proliferative and migrating/invasive potentials were further evaluated with the help of a CCK-8 and Transwell assays, respectively. Gain- and loss-of-function assays were also implemented to assess the role of miR-331-3p, SCARA5, and FAK pathway in PC cells. Lastly, xenograft nude mice were established to investigate the role of miR-331-3p *in vivo*. miR-331-3p negatively targeted SCARA5 and was highly expressed in CAFs-derived EVs, which accelerated the proliferative, migrating, and invasive potentials of PC cells. Meanwhile, over-expression of miR-331-3p enhanced the proliferative, migrating, and invasive properties of PC cells and promoted tumor growth *in vivo* by manipulating SCARA5/FAK axis, whereas SCARA5 countered the oncogenic effects of miR-331-3p. Overall, miR-331-3p in CAFs-derived EVs inhibits SCARA5 expression and activates the FAK pathway, thereby augmenting the progression of PC. Our study provides a potential therapeutic target for the treatment of PC.

## Introduction

Pancreatic cancer (PC) is one of the most common malignancies, characterized by poor prognoses, increased mortality in both males and females, and high mortality rates across the world.^1,2^ PC therapies include applications in surgery, radiotherapy, chemotherapy, and palliative care.^3^ However, the complexity of this malignancy and cumbersome early diagnoses impede the outcome and prognosis.^4^ Thus, in order to improve treatment of PC, a deeper understanding of the molecular mechanism and identification of specific druggable markers at genomic, epigenetic, and metabolic levels would go a long way in improving the quality of life of patients afflicted by PC.

Recently, a plethora of reports have implicated extracellular vesicles (EVs) in the tumorigenesis and chemoresistance of PC.^5–7^ EVs are characterized as heterogeneous, round or oval shaped, membrane-bound vesicles, which can be released by carcinoma-associated fibroblasts (CAFs) and take part in numerous biological processes including tumor development, metastasis, and chemoresistance.^8–12^ Moreover, EVs possess the ability to function as cargos to transport macromolecules, such as microRNAs (miRNAs), long noncoding RNAs (lncRNAs), mRNAs, proteins, and lipids, from donor cells into the extracellular matrix and recipient cells.^13,14^ Meanwhile, another molecule of great interest, miRNAs, are known to be small, endogenous non-coding RNAs that are approximately 20–22 nucleotides in length.^15^ These miRNAs function in a manner by resembling the RNA-induced silencing complex (RISC) and targeting the 3’-UTR of mRNAs, resulting in silencing of the gene expression.^16^ miRNAs have been elaborated to assume roles in the development and progression of cancers, with some miRNAs even being previously highlighted as potential therapeutic targets as well.^17–20^ For instance, microRNA-331-3p (miR-331-3p) was shown to enhance the proliferation and metastasis in hepatocellular carcinoma and breast cancer.^21,22^ However, in renal cell carcinoma and ovarian cancer, miR-331-3p contributes to the suppression of tumorigenesis.^23,24^ Interestingly, in the context of PC, miR-331-3p it has been previously implicated as an oncogene.^25^ However, the underlying molecular mechanism of how miR-331-3p triggered PC development is not fully understood.

In lieu of this, the current research performed bioinformatics analyses to identify miR-331-3p expression in human PC and its downstream targets. In addition, we performed cellular, histological, molecular approaches, and *in vivo* experiments, we set out to elucidate the role of miR-331-3p in PC development and decipher the molecular mechanism both *in vitro* and *in vivo*, hoping to shed light on a novel strategy for therapeutic strategies against PC.

## Results

### CAFs-derived EVs promote the proliferative, migratory, and invasive abilities of PC cells

**Figure 1.**
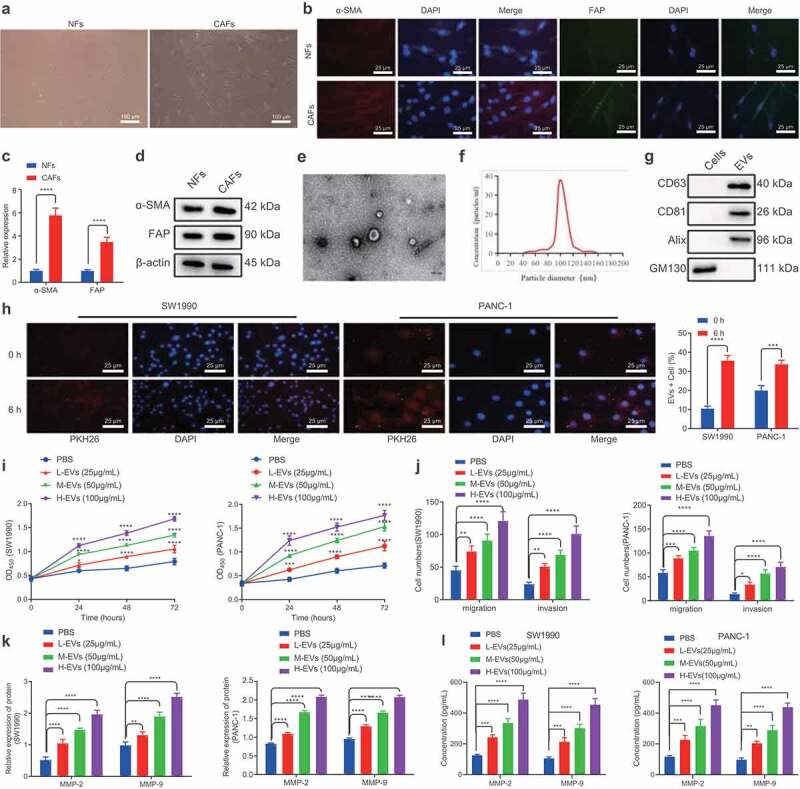
CAFs-derived EVs promote the proliferation, migration and invasion of PC cells.

First, to study the effect of CAFs-derived EVs on PC, we collected CAFs and normal fibroblasts (NFs) from PC and corresponding adjacent normal tissues. Subsequent observation under an inverted microscope revealed that CAFs were spindle-shaped, while NFs cells were in the shape of flattened stars with a radial appearance (Figure 1(a)). Meanwhile, immunofluorescence illustrated that α-SMA and FAP were highly expressed in CAFs, while being poorly- or barely expressed in NFs (Figure 1(b)). In addition, RT-qPCR and Western blot analysis showed that α-SMA and FAP expressions in CAFs were substantially higher than those in NFs (Figure 1(c,d)), which confirmed the successful isolation of CAFs and NFs. Next, we isolated and purified the EVs generated by CAFs and NFs, which were observed as a group of heterogeneous, round, or oval shaped, membrane-bound vesicles, wherein the membranous structure could be seen on the periphery of the vesicles (Figure 1(e), Supplementary Figure 1A). Nanosight nanoparticle tracking analyses further uncovered that EVs exhibited an irregular Brownian motion with a diameter between 40 and 160 nm (Figure 1(f), Supplementary Figure 1B). Moreover, EV marker proteins, CD63, CD81, and Alix, were all positively expressed, while GM130 was not expressed (Figure 1(g), Supplementary Figure 1C). Furthermore, PKH26-labeled EVs were co-cultured with SW1990 and PANC-1 cells for 6 h, and observation under a fluorescence microscope revealed that the recipient cells (SW1990 and PANC1) showed high EV uptake efficiency (Figure 1(h)). Overall, these findings demonstrated the successful isolation of EVs, and EVs derived from CAFs could be internalized by PC cells.

A. Inverted microscopic images of the morphologies of CAFs and NFs. B. IF detection of the expression of α-SMA and FAP in CAFs and NFs. C. Expression of α-SMA and FAP mRNAs in CAFs and NFs determined by RT-qPCR. D. Expression of α-SMA and FAP proteins in CAFs and NFs determined by Western blot analysis. E. TEM visualization of the morphology of CAFs-derived EVs. F. Size distribution of the CAFs-derived EVs assessed by NTA. G. Expression of EVs-related proteins determined by Western blot analysis. H. Internalization of EVs by SW1990 and PANC-1 cells under a confocal microscope. Blue indicates DAPI staining and rad indicates PKH67-labeled EVs. I. The proliferation of SW1990 and PANC-1 cells after co-culture with CAFs-derived EVs for 48 h measured by CCK-8 assay. J. Migration and invasion of SW1990 and PANC-1 cells after co-culture with CAFs-derived EVs for 48 h measured by Transwell assay. K. Expression of tumor-related proteins MMP-2 and MMP-9 in SW1990 and PANC-1 cells after co-culture with CAFs-derived EVs for 48 h determined by Western blot analysis. L. Expression of MMP-2 and MMP-9 in the supernatant of SW1990 and PANC-1 cells following co-culture with EVs for 48 h measured by ELISA. Experiments were repeated 3 times. ** indicates *p* < .01, *** indicates *p* < .001, and **** indicates *p* < .0001.

Subsequently, we aimed to identify the effects of CAFs-derived EVs on PC cells. Increasing concentrations of EVs were found to promote the proliferative, migratory, and invasive capabilities of SW1990 and PANC-1 cells (Figure 1(i,j)). In addition, following co-culture with EVs for 48 h, SW1990 and PANC-1 cells were observed to be present with up-regulated MMP-2 and MMP-9 levels (Figure 1(k), Supplementary Figure 2A). Meanwhile, ELISA conducted on the supernatant of SW1990 and PANC-1 cells were consistent with those of Western blot analysis (Figure 1(l)). Both cell lines SW1990 and PANC1 were further treated with NFs-derived EVs, and the results shown in Supplementary Figure 1D-F illustrated that NFs-derived EVs exerted no significant effects on the proliferative, migrating, and invasive properties of SW1990 and PANC-1 cells, as well as on MMP-2 and MMP-9 expression. Taken together, CAFs-derived EVs could promote the proliferative, migratory, and invasive potentials of PC cells.

### SCARA5 is poorly expressed in PC tissues and further associated with poor prognoses in PC patients

**Figure 2.**
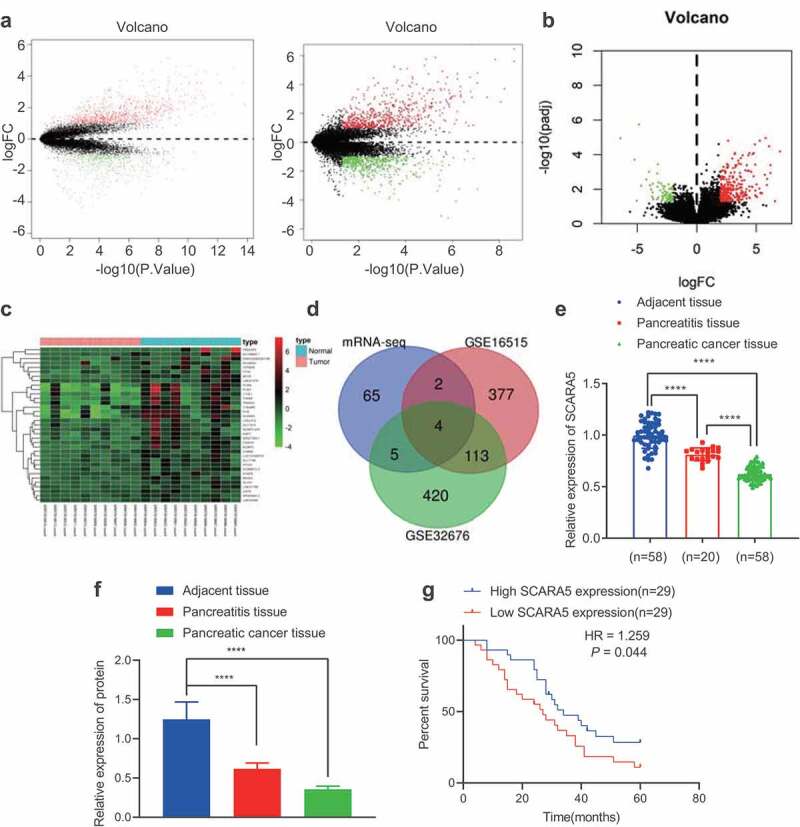
SCARA5 is poorly expressed in PC.

The GEO database was adopted to obtain human PC-related mRNA expression datasets, GSE16515 and GSE32676, and differentially expressed mRNAs were filtered with |*logFC| > 1 and p value < .05*. We subsequently came across 835 up-regulated and 496 down-regulated mRNAs from the GSE16515 dataset, and 646 up-regulated and 542 down-regulated mRNAs from the GSE32676 dataset (Figure 2a). The transcriptomic sequencing data of human PC and corresponding normal tissues were additionally retrieved from the SRA database including SRR7818914, SRR7818913, RR7818912, SRR7818911, SRR7818910, SRR7818909, SRR7818908, SRR7818907, SRR7818906, SRR7818905, SRR7818904, SRR7818903, SRR7818902, SRR7818901, SRR7818900, SRR7818899, SRR7818898, SRR7818897, SRR7818896, and SRR7818895. Next, the R package “DESeq2” was employed to analyze the differentially expressed genes filtered with |*logFC| > 2 and adjusted p value < .01*, which revealed a total of 283 up-regulated and 76 down-regulated mRNAs (Figure 2b). The top 35 appreciably down-regulated mRNAs in human PC tissues were visualized (Figure 2c) and then intersected with the strikingly down-regulated genes in the GSE16515 and GSE32676 datasets from the GEO database. A total of 4 genes (SCARA5, PLIN4, LYVE1, and ADH1B) were found at the intersection (Figure 2d). Among these four genes, SCARA5 was the most profoundly down-regulated in PC tissues (Figure 2c). RT-qPCR was then adopted to determine the expression patterns of SCARA5 in PC tissues, adjacent normal tissues, and pancreatitis tissues, which revealed that SCARA5 expression was remarkably down-regulated in PC and pancreatitis tissues relative to adjacent normal tissues; as compared to pancreatitis tissues, SCARA5 expression was markedly decreased in PC tissues (Figure 2e). Moreover, Western blot analysis results on SCARA5 expression were identical to those of RT-qPCR (figure 2f, Supplementary Figure 2B). Furthermore, Kaplan–Meier curve analysis manifested that the overall survival of PC patients with low SCARA5 expression was prominently worse than that of patients with high SCARA5 expressions (Figure 2g). Altogether, SCARA5 was poorly expressed in PC tissues, and further associated with poor prognoses in PC patients.

A. The volcano plot of mRNA expression from GSE16515 and GSE32767 PC expression datasets. The black dot indicates mRNAs without significant difference, the green dot represents significantly downregulated mRNAs, and the red dot shows significantly upregulated mRNAs in PC. B. The volcano plot of mRNA expression sequencing data obtained from SRA database, the black dot indicates mRNAs without significant difference. The green dot represents significantly downregulated mRNAs, and the red dot shows significantly upregulated mRNAs in PC. C. A heat map of differential analysis to show the top 35 most significantly downregulated mRNAs in PC. The x-axis presents the sample number and the y-axis indicates the mRNAs. Each small square in the figure indicates the expression of an mRNA in a sample. D. Intersected significantly downregulated mRNAs in PC obtained from SRA database and from GSE16515 and GSE32767 PC expression datasets. Blue circle indicates significantly downregulated genes *via* differential analysis of PC transcriptomic sequencing data, red circle indicates significantly downregulated genes *via* differential analysis of PC GSE16515 expression dataset, and green circle indicates significantly downregulated genes *via* differential analysis of PC GSE32676 expression dataset. The overlay represents the intersection of the three sets of data. E. The expression of SCARA5 in PC tissues, adjacent normal tissues, and pancreatitis tissues determined by RT-qPCR. F. The protein expression of SCARA5 in PC tissues, adjacent normal tissues, and pancreatitis tissues determined by Western blot analysis. G. Kaplan–Meier curve analysis of the correlation between SCARA5 expression and the overall survival of PC patients.

#### SCARA5 is a target gene of miR-331-3p in PC cells

**Figure 3.**
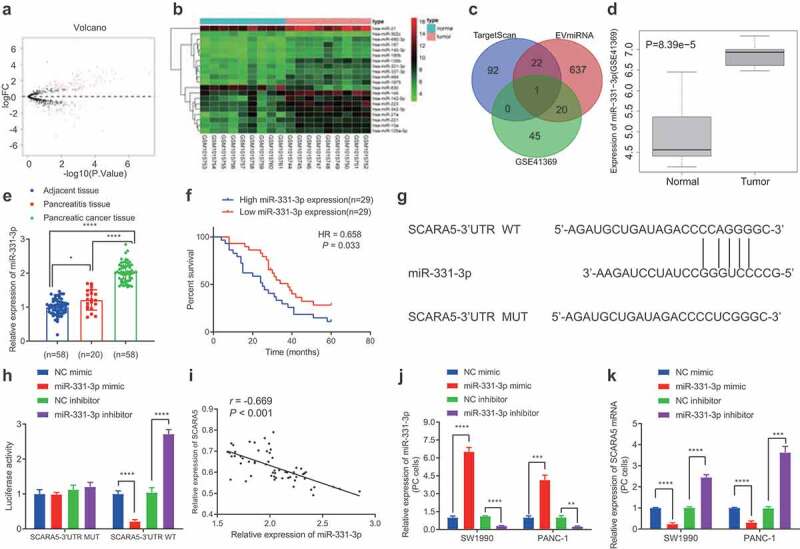
miR-331-3p targets SCARA5 in PC cells.

Numerous studies have indicated that SCARA5 is under-expressed in various malignancies, while SCARA5 deficiency is further known to promote cancer cell progression.^26,27^ To figure out the upstream miRNAs of SCARA5, we subsequently retrieved human PC-related miRNA expression dataset GS41369 from the GEO database, wherein the differentially expressed miRNAs were filtered with |*logFC| > 1 and p value < .05* as the screening criteria, which reared a total of 66 markedly up-regulated and 40 notably down-regulated miRNAs (Figure 3a). Next, we visualized the top 20 considerably up-regulated miRNAs in human PC samples (Figure 3b), which were intersected with the upstream miRNAs of SCARA5 predicted by the TargetScan and miRNAs in human fibroblast-derived EVs predicted by the EVmiRNA databases. It was found that only miR-331-3p was at this intersection (Figure 3c). Meanwhile, miR-331-3p was further illustrated to be up-regulated in the GSE41369 dataset (Figure 3d).

A. The volcano plot of miRNA expression from GSE41369 PC expression dataset. The black dot indicates miRNAs without significant difference, the green dot represents significantly downregulated miRNAs, and the red dot shows significantly upregulated miRNAs in PC. B. Heat maps of differential analysis from GSE41369 PC expression datasets to show the top 20 most significantly upregulated miRNAs. The x-axis presents the sample number and the y-axis indicates the miRNAs. Each small square in the figure indicates the expression of a miRNA in a sample. C. Prediction of upstream miRNAs of SCARA5. The three ellipses in the figure represent the prediction results of miRNAs from TargetScan and miRNAs in human fibroblast EVs from EVmiRNA database and significantly upregulated miRNAs in PC miRNA expression dataset GSE41369. The overlay represents the intersection of the three sets of data. D. The expression of miR-331-3p in PC expression dataset GSE41369. E. The expression of miR-331-3p in PC, corresponding to healthy and pancreatitis tissues. F. Kaplan–Meier analysis of the relationship between the survival of 58 PC patients and expression of miR-331-3p. G. The binding sites between SCARA5 and miR-331-3p predicted by TargetScan. H. Dual-luciferase reporter assay to assess the binding of miR-331-3p to SCARA5. I. Pearson’s correlation coefficient analysis of the association between miR-331-3p and SCARA5 expression in 58 PC tissues. J. Expression miR-331-3p in SW1990 and PANC-1 cells after transfection determined by RT-qPCR. K. Expression of SCARA5 in SW1990 and PANC-1 cells after transfection determined by RT-qPCR. Experiments were repeated 3 times. * indicates *p* < .05 and *** indicates *p* < .001.

Meanwhile, studies have also reported that up-regulated levels of miR-331-3p promote the tumor growth and metastasis in human PC.^25,28^ We performed an RT-qPCR assay to determine the expression patterns of miR-331-3p in human PC tissues, and the results of which demonstrated that miR-331-3p levels were noticeably higher in human PC tissues compared to those in corresponding normal tissues and pancreatitis tissues (Figure 3e). In addition, Kaplan–Meier analysis revealed that the survival rate of PC patients with high expression of miR-331-3p was observably lower compared with patients exhibiting low expressions of miR-331-3p (figure 3f). Moreover, based on the potential binding sites between miR-331-3p and SCARA5 (Figure 3g), a dual-luciferase reporter assay was performed for validation, and the subsequent results demonstrated that miR-331-3p mimic dramatically diminished the luciferase activity in SCARA5-3’-UTR-WT, whereas miR-331-3p inhibitor exerted the contrary effect. However, luciferase activity in SCARA5-3’-UTR-MUT was found to be unaltered after treatment with miR-331-3p mimic or miR-331-3p inhibitor (Figure 3h). Pearson’s correlation coefficient and further data suggested that miR-331-3p was adversely associated with SCARA5 in the PC tissues (Figure 3i). Additionally, we transduced miR-331-3p mimic or inhibitor in SW1990 and PANC-1 cells, and 24 h after lentiviral transduction demonstrated that miR-331-3p expression was considerably increased, while the SCARA5 expression was markedly reduced as a result of miR-331-3p mimic treatment, whereas the conflicting trends were observed after miR-331-3p inhibition (Figure 3j, k). Overall, miR-331-3p may target SCARA5 and inhibit its expression in PC cells.

#### CAFs-derived EVs transfer miR-331-3p to suppress SCARA5 expression in PC cells

**Figure 4.**
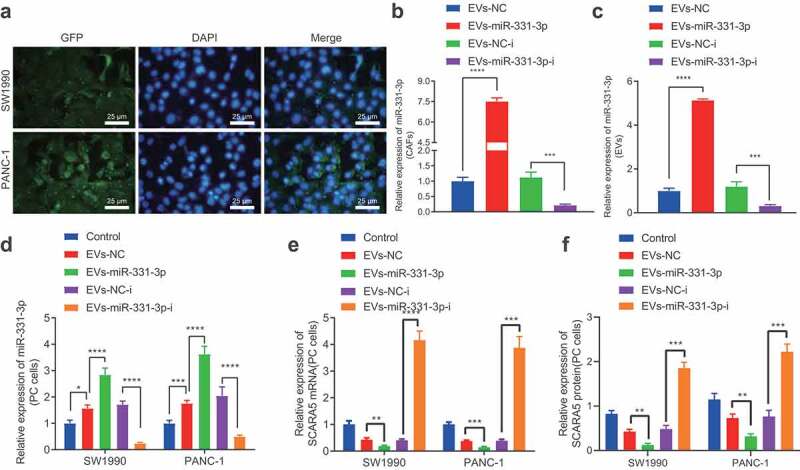
CAFs-derived EVs transport miR-331-3p to inhibit the expression of SCARA5.

To further elucidate the mechanism by which CAFs contribute to the development of PC, we determined the levels of miR-331-3p in CAFs and NFs, as well as PC cells SW1990 and PANC-1 with the help of qPCR (Supplementary Figure 3). It was found that miR-331-3p levels were significantly higher in CAFs compared to SW1990 and PANC-1 cells and NFs while also being elevated in SW1990 and PANC-1 cells than those in NFs. Next, CAFs transfected with miR-331-3p-Cy3 were co-cultured with PC cells transfected with pCDNA3.1-GFP, and observed under a laser scanning confocal microscopy to ascertain the uptake of EVs by PC cells. PC cells emitted red fluorescence (Figure 4a). The EVs were subsequently isolated from CAFs before and after transduction, and RT-qPCR displayed that miR-331-3p expression was remarkably up-regulated in the CAFs and the derived EVs following miR-331-3p treatment, while contrary results were observed in the presence of miR-331-3p-i (Figure 4b, c). Then, the isolated EVs were co-cultured with SW1990 and PANC-1 cells, and miR-331-3p expression was up-regulated, but SCARA5 mRNA was down-regulated in SW1990 and PANC-1 cells co-cultured with EVs, especially with EVs-miR-331-3p, while miR-331-3p expression was found to be notably down-regulated, but SCARA5 was up-regulated in SW1990 and PANC-1 cells co-cultured with EVs-miR-331-3p-i (Figure 4d, e). Western blot analysis was further conducted for verification (figure 4f), SCRA5 expression was considerably decreased in SW1990 and PANC-1 cells co-cultured with EVs-miR-331-3p, while the SCARA5 expression was notably increased in SW1990 and PANC-1 cells co-cultured with EVs-miR-331-3p-i. Altogether, CAFs-derived EVs transport miR-331-3p to suppress SCARA5 expression in PC cells.

A. PC cells transfected with pCDNA3.1-GFP following co-culture with CAFs transfected with miR-331-3p-Cy3 observed under a laser scanning confocal microscopy. B. miR-331-3p expression in CAFs following miR-331-3p overexpression or inhibition determined by RT-qPCR. C. miR-331-3p expression in EVs isolated from CAFs following miR-331-3p overexpression or inhibition determined by RT-qPCR. D, miR-331-3p expression in SW1990 and PANC-1 cells before and after co-culture with EVs determined by RT-qPCR. E. SCARA5 expression in SW1990 and PANC-1 cells after co-culture with EVs-miR-331-3p or EVs-miR-331-3p-i for 48 h determined by RT-qPCR. F. Expression of SCARA5 in SW1990 and PANC-1 cells after co-culture with EVs-miR-331-3p or EVs-miR-331-3p-i for 48 h determined by Western blot analysis. Experiments were repeated 3 times. ** indicates *p* < .01, *** indicates *p* < .001, and **** indicates *p* < .0001.

***CAFs-derived EVs transport miR-331-3p to accelerate the proliferative, migratory and invasive abilities of PC cells***

**Figure 5.**
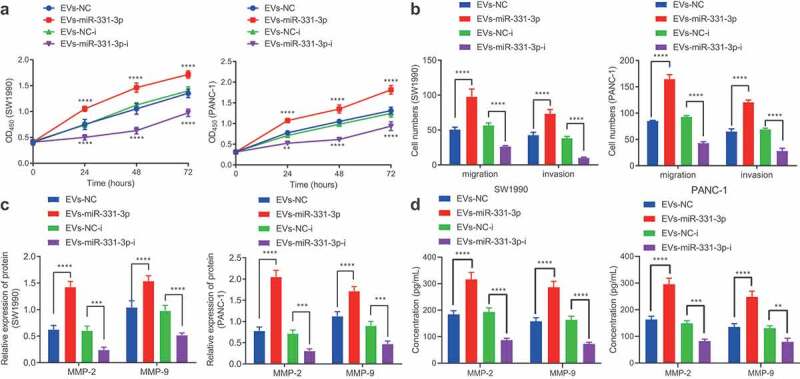
CAFs-derived EVs transport miR-331-3p to promote the proliferation, migration and invasion of PC cells.

Additionally, we explored the effects of miR-331-3p delivered by CAFs-derived EVs on PC cells. It was found that the proliferative (Figure 5a), migratory, and invasive (Figure 5b) potentials and MMP-2 and MMP-9 expressions (Figure 5c) in SW1990 and PANC-1 cells were all enhanced following co-culture with EVs-miR-331-3p. In contrast, the contrary trends were observed in SW1990 and PANC-1 cells co-cultured with EVs-miR-331-3p-i (Figure 5a-C, Supplementary Figure 2C). Furthermore, ELISA detection results of MMP-2 and MMP-9 expression patterns in the supernatant of SW1990 and PANC-1 cells were similar to those of Western blot analysis (Figure 5d). Overall, miR-331-3p transferred by CAFs-derived EVs could promote the proliferative, migratory, and invasive abilities of PC cells.

A. Proliferation of SW1990 and PANC-1 cells after co-culture with EVs-miR-331-3p or EVs-miR-331-3p-i measured by CCK-8 assay. B. Migration and invasion of SW1990 and PANC-1 cells after co-culture with EVs-miR-331-3p or EVs-miR-331-3p-i for 48 h measured by Transwell assay. C. Expression of tumor-related proteins in SW1990 and PANC-1 cells after co-culture with EVs-miR-331-3p or EVs-miR-331-3p-i for 48 h determined by Western blot analysis. D. Expression of MMP-2 and MMP-9 in the supernatant of SW1990 and PANC-1 cells following co-culture with EVs-miR-331-3p or EVs-miR-331-3p-i for 48 h measured by ELISA. Experiments were repeated 3 times. ** indicates *p* < .01, *** indicates *p* < .001, and **** indicates *p* < .0001.

***miR-331-3p delivered by CAFs-derived EVs inhibits SCARA5 expression and further augments the proliferative, migratory and invasive abilities of PC cells***

**Figure 6.**
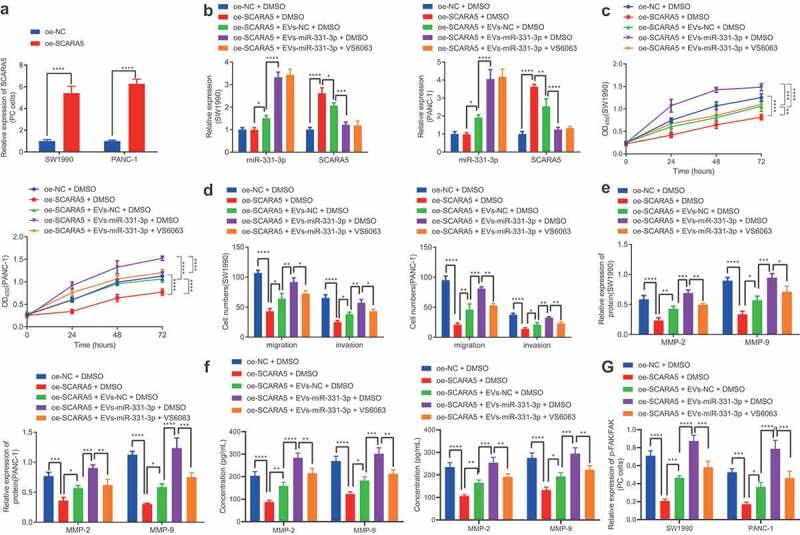
CAFs-derived EVs transport miR-331-3p to promote the proliferation, migration and invasion of PC cells by suppressing SCARA5.

In an effort to investigate whether CAFs-derived EVs transport miR-331-3p to regulate SCARA5 expression resulting in PC, we over-expressed SCARA5 in SW1990 and PANC-1 cells. Subsequent results of RT-qPCR demonstrated that SCARA5 expression was significantly elevated in SW1990 and PANC-1 cells over-expressing SCARA5 (Figure 6a). Meanwhile, existing evidence suggests that SCARA5 inhibits the FAK pathway to suppress tumor proliferation and metastasis,^29^ whereas down-regulation of FAK pathway is also known to inhibit cell migrating and invasive abilities in PC,^30^ which suggests that SCARA5 may affect cell migration and invasion of PC through the FAK pathway. Accordingly, we co-cultured EVs isolated from CAFs mimicking miR-331-3p expression with SW1990 and PANC-1 cells over-expressing SCARA5 or treated with VS6063. RT-qPCR exhibited that oe-SCARA5 did not alter the miR-331-3p expression but enhanced that of SCARA5 in SW1990 and PANC-1 cells in the presence of DMSO. On the other hand, in the presence of oe-SCARA5 + DMSO, miR-331-3p was considerably increased, while the SCARA5 expression was remarkably restrained by co-culture with EVs-NC. In addition, a pronounced increase in miR-331-3p expression and a significant decrease in SCARA5 expression were noted in SW1990 and PANC-1 cells following co-culture with EVs-miR-331-3p in contrast to co-culturing with EVs-NC in the presence of oe-SCARA5 + DMSO. Moreover, no alterations were documented in the miR-331-3p and SCARA5 expressions of SW1990 and PANC-1 cells between treatments with oe-SCARA5 + EVs-miR-331-3p + DMSO and oe-SCARA5 + EVs-miR-331-3p + VS6063 (Figure 6b).

A. The expression of SCARA5 in SW1990 and PANC-1 cells overexpressing SCARA5 determined by RT-qPCR. SW1990 and PANC-1 cells were treated with oe-SCARA5 + DMSO, oe-SCARA5 + EVs-NC + DMSO, oe-SCARA5 + EVs-miR-331-3p + DMSO or oe-SCARA5 + EVs-miR-331-3p + VS6063. B. The expression of miR-331-3p and SCARA5 in SW1990 and PANC-1 cells determined by RT-qPCR. C. Proliferation of SW1990 and PANC-1 cells measured by CCK-8 assay. D. Migration and invasion of SW1990 and PANC-1 cells measured by Transwell assay. E. Expression of MMP-2 and MMP-9 in SW1990 and PANC-1 cells determined by Western blot analysis. F. Expression of MMP-2 and MMP-9 in the supernatant of SW1990 and PANC-1 cells measured by ELISA. G. p-FAK/FAK ratio in SW1990 and PANC-1 cells determined by Western blot analysis. Experiments were repeated 3 times. ** indicates *p* < .01, *** indicates *p* < .001, and **** indicates *p* < .0001.

Furthermore, the proliferative (Figure 6c), migratory, and invasive (Figure 6d) abilities of SW1990 and PANC-1 cells, MMP-2 and MMP-9 expression (Figure 6e, f), as well as the ratio of p-FAK/FAK (Figure 6g) were all found to be significantly diminished by SCARA5 over-expression in the presence of DMSO, which was nullified by EVs-NC. In addition, Evs-miR-331-3p were observed to augment the proliferative, migratory, and invasive abilities of SW1990 and PANC-1 cells, MMP-2 and MMP-9 expressions, and the ratio of p-FAK/FAK in comparison to EVs-NC in the presence of oe-SCARA5 + DMSO group. Moreover, downward trends were noted in the proliferative, migratory, and invasive abilities of SW1990 and PANC-1 cells, MMP-2 and MMP-9 expression, and the ratio of p-FAK/FAK after treatment with oe-SCARA5 + EVs-miR-331-3p + VS6063 as compared with treatment with oe-SCARA5 + EVs-miR-331-3p + DMSO (Figure 6c-G, Supplementary Figure 2D). In summary, CAFs-derived EVs-enclosed miR-331-3p target and inhibit SCARA5, thus stimulating the proliferation, migration, and invasion of PC cells.

#### CAFs-derived EVs transport miR-331-3p to promote the tumorigenesis of PC in vivo

**Figure 7.**
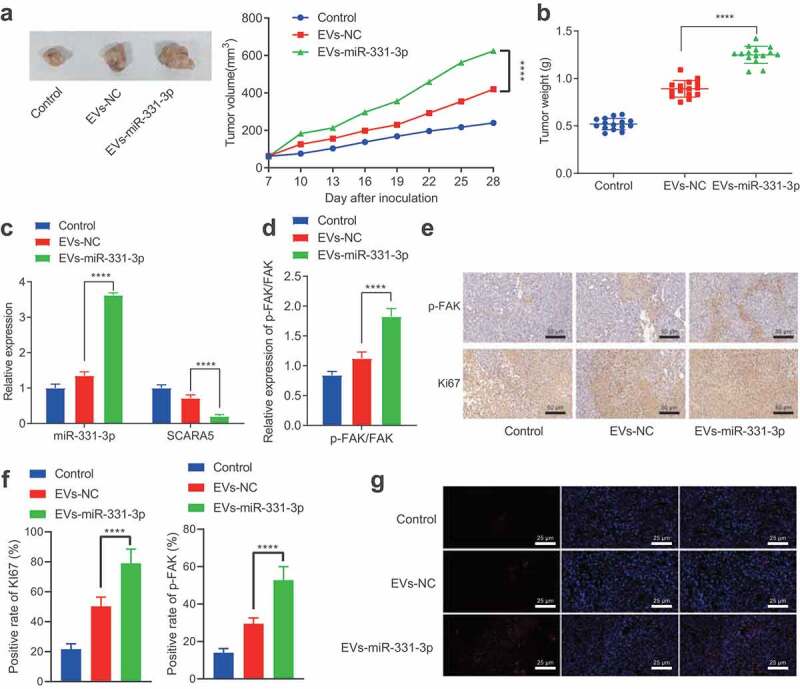
CAFs-derived EVs transport miR-331-3p to promote the tumorigenesis of PC *in vivo.*

Lastly, to investigate whether miR-331-3p delivered by CAFs-derived EVs could promote the tumorigenesis of PC *in vivo*, we transplanted PANC-1 cells into nude mice subcutaneously to establish xenograft mouse models, which were then injected with CAFs-derived EVs. Tumor volume and weight were both found to be notably enhanced by EVs-NC and EVs-miR-331-3p, especially by EVs-miR-331-3p (Figure 7a, b). Subsequent results revealed that miR-331-3p expression and p-FAK/FAK ratio were notably elevated, while the SCARA5 expression was remarkably diminished after treatment with EVs-NC and EVs-miR-331-3p, especially after EVs-miR-331-3p treatment (Figure 7c, d). Moreover, immunohistochemistry illustrated that the phosphorylation levels of FAK and proliferation-related factor Ki67 expression were augmented by EVs-NC and EVs-miR-331-3p, especially by EVs-miR-331-3p (Figure 7e, f). Ki67 and p-FAK were further observed to be primarily located in the cell (Figure 7e). In addition, the proportion of miR-331-3p fluorescence-positive cells in the EVs-miR-331-3p group was evidently higher than that in the EVs-NC group (Figure 7g). Altogether, CAFs-derived EVs transport miR-331-3p, which inhibits SCARA5 and activates FAK to promote the tumorigenesis of PC *in vivo*.

A. Representative images showing xenografts in nude mice and tumor volume statistics. B. The graph of tumor weight. C. Expression of miR-331-3p and SCARA5 in mouse tumor tissues determined by RT-qPCR. D. Expression of proteins in FAK pathway in mouse tumor tissues determined by Western blot analysis. E, Representative images of Ki67 and p-FAK in mouse tumor tissues determined by immunohistochemistry. F, Quantitative analysis of panel E. G, Fluorescence probe detection of the cellular localization of miR-331-3p. ** indicates *p* < .01, *** indicates *p* < .001, and **** indicates *p* < .0001. n = 15 for mice in each group.

## Discussion

PC is one of the leading causes of cancer-related mortality worldwide; however, the prevention and early diagnosis of the aggressive carcinoma are challenging.^31^ It would be prudent to better understand the underlying mechanism of this disease to identify molecular biomarkers to advance the diagnostic and treatment strategies against PC.

Our obtained findings revealed that CAFs-derived EVs can be up-taken by PC cells to promote the cell proliferation, migration, and invasion. Inherently, CAFs-derived EVs are known to play critical roles during the communication between cells and the microenvironment, and further possess the ability to transport miRNAs to cancer cells regulating cancer progression processes, such as cell proliferation and metastasis.^32^ Meanwhile, bioinformatics analyses in our study illustrated the presence of miR-331-3p in CAFs-derived EVs and the high expression of miR-331-3p in pancreatic tissues, which was further correlated with poor prognoses in PC patients. In addition, the co-culture of isolated EVs from CAFs and PC cell lines validated that miR-331-3p can be transported from EVs into PC cells. Furthermore, we adopted gain- and loss-of-function experiments, which demonstrated that miR-331-3p promotes the proliferation, migration, and invasion of PC cells, and also augments the expression of cancer cell metastasis markers, MMP-2 and MMP-9. Collectively, these findings make it plausible to suggest that CAFs-derived EVs exert their oncogenic functions through miR-331-3p in PC.

It is also important to recognize the hard-done work of our peers, which has elucidated that miRNAs function as regulators of gene expression by interfering the stability and translation of mRNAs.^33,34^ Not only implicated in normal biological processes in cells, miRNAs have also been previously shown to play an important role in the regulation of cancer development including PC.^35–39^ To elaborate, the study performed by *Chen et al*. revealed that miR-331-3p was notably increased in pancreatic patient samples cell lines and down-regulation of miR-331-3p suppressed cell proliferation and metastasis *in vitro*, which is in accordance with our findings.^25^ However, the mechanism behind miR-331-3p inducing the progression of PC and downstream targets of miR-331-3p remains elusive. As a result, we adopted a bioinformatics analysis and a dual-luciferase reporter assay, and the findings of which uncovered SCARA5 as a direct target of miR-331-3p, which is also poorly expressed in PC. Scavenger receptors belong to a family of membrane receptors that are expressed on the surface of monocytes and macrophages.^40^ More specifically, SCARA5, a novel member of the scavenger receptor family, is known to be expressed by epithelial cells.^41^ Moreover, *Yan et al*. illustrated that up-regulation of SCARA5 inhibits tumor growth and metastasis, which sheds a light on the anti-tumor effect of SCARA5.^42^ Expanding on this, our findings further revealed that miR-331-3p in CAFs-derived EVs targets and inhibit of SCARA5 expression, while gain- and loss-of-function studies and functional analyses indicated that miR-331-3p suppresses the SCARA5 expression and promotes the proliferation, migration, and invasion of PC. Altogether, these findings and data provide enough evidence to highlight SCARA5 as a tumor suppressor.

Additionally, SCARA5 is also known to be capable of suppressing tumor proliferation and invasion by inhibiting the FAK pathway.^29,43^ The FAK pathway is responsible for the cell migration and angiogenesis, while repression of the FAK pathway can inhibit cell motility and proliferation.^44,45^ Moreover, a plethora of studies have previously indicated inhibition of FAK as a therapeutic strategy against PC,^46–48^ which also roused us to explore whether miR-331-3p in CAFs-derived EVs promotes the development of PC by regulating the SCARA5/FAK axis. Subsequent findings in our study demonstrated that inhibition of FAK by its inhibitor VS6063 could significantly decrease the levels of p-FAK/FAK in FAK pathway, metastasis markers MMP-2 and MMP-9, and further remarkably suppress the progression of PC cells. Lastly, we also established pancreatic tumor-bearing xenograft nude mice to validate the oncogenic role of miR-331-3p *in vivo*, and our data revealed that miR-331-3p considerably increases the tumor size, volume, and weight in nude mice and promotes the tumorigenesis of PC through SCARA5/FAK axis *in vivo*.

**Figure 8.**
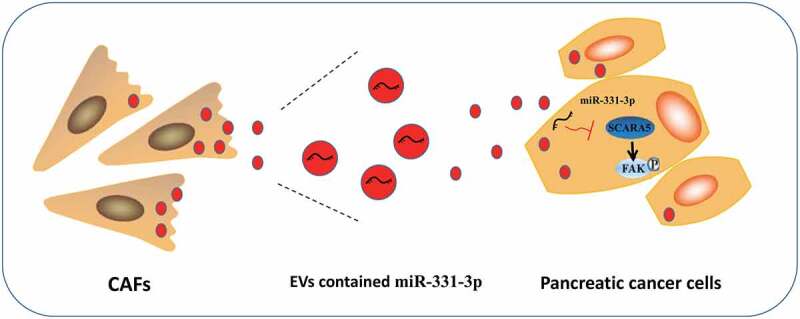
Schematic diagram of the mechanism by which miR-331-3p delivered by CAFs-derived EVs affect PC.

Altogether, the findings obtained in our study highlight a novel downstream target, SCARA5, of miR-331-3p and elaborate on the underlying molecular mechanism that miR-331-3p in CAFs-derived EVs exerts its oncogenic function by regulating the SCARA5/FAK axis in PC (Figure 8), which can be harnessed in the future as a novel therapeutic strategy for the treatment of PC. However, the side effects of targeting miR-331-3p and/or FAK pathway remain to be evaluated, and further investigations are warranted to validate the feasibility of this therapeutic strategy to increase the quality of life of patients affected by PC.

CAFs-derived EVs transfer miR-331-3p into PC cells where miR-331-3p inhibits SCARA5 expression to activate FAK pathway, enhancing the proliferation, migration, and invasion of PC cells *in vitro* and promoting the tumor growth *in vivo*, ultimately contributing to the progression of PC.

## Methods

### Ethics statement

The current research was ratified by the Ethics Committee of The First Affiliated Hospital of Soochow University and implemented strictly following the *Declaration of Helsinki*. Signed informed consents were offered by all participants before sample collection. The animal experimental processes were approved by the Ethics Committee of The First Affiliated Hospital of Soochow University and conducted in strict accordance with the Guide for the Care and Use of Laboratory Animals.

### Bioinformatics analysis

First, SRA database was employed to obtain transcriptomic sequencing data of human PC tissues and corresponding normal tissues, which was filtered by |*logFC| > 2 and adjusted p value < .01*, and analyzed with the R package “DESeq2” to get differentially expressed genes. Additionally, the GEO database was adopted to obtain the gene expression data of human PC including GSE16515, GSE32676, and GSE41369 filtered using |*logFC| > 1 and adjusted p value < .05*, which were again processed with the R language “limma” package to analyze the differentially expressed genes. Subsequently, a Venn diagram was plotted to analyze conspicuously down-regulated mRNAs screened by the SRA database through differential expression analysis of transcriptomic sequencing data of human PC and the GEO database *via* analysis of the GSE16515 and GSE32676 expression datasets to determine the target genes. Furthermore, the upstream miRNA of the SCARA5 gene was predicted using TargetScan, and miRNA in human fibroblast EVs was obtained through the EVmiRNA database. Finally, the notably up-regulated miRNAs filtered from the GSE41369 expression dataset (padj = 0.05, logFC = 1) were intersected with data predicted by the TargetScan and EVmiRNA databases to determine the target miRNA.

### Tissue samples

A total of 58 PC tissue samples and corresponding adjacent normal tissue samples or 20 pancreatitis tissue samples that were excised and confirmed by pathology were collected from patients receiving surgical resection between September 2012 and September 2014 at The First Affiliated Hospital of Soochow University. None of the patients underwent radiotherapy, chemotherapy, or biological therapy before surgery, and all patients were subjected to radical surgery. The attained samples were flash-frozen in liquid nitrogen and stored at −80°C for subsequent experimentation. Patient samples were obtained from 41 males and 17 females, with an average of 61 years old (age range 48–72 years). Outpatient data survey, outpatient follow-up, and telephonic follow-up were carried out to ensure patients check pancreas-specific antigen (PaA) once a month for the first 3 months after discharge, and PaA and digital rectal examination (DRE) every 3 months thereafter. The review was conducted every 6 months after 2 years of operation, and PaA was checked every year after 5 years of operation. The follow-up time of our study was from diagnosis to the patients’ death, around 4–60 months. The Kaplan–Meier method was adopted to analyze the patients’ survival rate.

### Isolation of CAFs and normal fibroblasts (NFs)

PC and normal tissue samples were sliced into 1 mm^3^ cubes before 2-h digestion with 1 mg/mL collagenase I (#C0130, Sigma-Aldrich, St Louis, MO) at 37°C. EV-free Dulbecco’s modified Eagle’s medium (DMEM) encompassing 10% fetal bovine serum (FBS; A2720801, Gibco, Gaithersburg, MD, with EVs removed) and 3% penicillin and streptomycin (Gibco, Gaithersburg, MD) were then added to neutralize collagenase before 5-min centrifugation at 500 g and 4°C and filtration through a 100 μm filter. Next, the isolated cells underwent culture with DMEM/F12 medium encompassing 10% FBS, 1% penicillin (Life Technologies, Carlsbad, CA), and 1% streptomycin (Life Technologies, Carlsbad, CA) in a 37°C and 5% CO_2_ incubator. The medium was changed after 3–5 d and every 3 d thereafter. Subsequent to 14 days, cells were observed to fully-cover the petri dish. Upon reaching an 80–90% confluency, the cells were trypsinized and passaged with a rate of 1: 3. CAFs and NFs utilized for further experimentation were the 3rd and 5th passages of cells cultured *in vitro*, respectively. The CAFs were identified by means of cell morphology observation, immunofluorescence (IF), and Western blot analysis.

### Cell culture

Human PC cell lines SW1990 (CRL-2172) and PANC-1 (CRL-1469) were procured from American Type Culture Collection ([ATCC], Manassas, VA). Next, the CAFs, SW1990, and PANC-1 cells received culture in EV-free DMEM encompassing 10% FBS (A2720801, Gibco, with EVs removed), 1% penicillin, and 1% streptomycin in a humidified incubator with 5% CO_2_ at 37°C (Thermo Fisher Scientific, Waltham, MA). Following culture, the medium was removed, followed by digestion with 0.25% trypsin for 2–5 min. Afterward, the CAFs, SW1990, and PANC-1 cells were resuspended, passaged, and cultured with 5 mL EVs-free DMEM encompassing 10% FBS (with EVs removed), 1% penicillin, and 1% streptomycin.

## Cell treatment

CAFs were transduced with lentivirus and grouped into the following groups: the negative control (NC), the miR-331-3p, the NC-inhibitor (NC-i), and the miR-331-3p-i groups. Meanwhile, SW1990 and PANC-1 were transfected and grouped into the following groups: the NC mimic, the miR-331-3p mimic, the NC inhibitor, the miR-331-3p inhibitor, the overexpression (oe)-NC, oe-SCARA5 (GenePharma, Shanghai, China), the miR-331-3p mimic + oe-NC, the miR-331-3p mimic + oe-SCARA5, the dimethyl sulfoxide (DMSO), and the VS6063 groups (focal adhesion kinase [FAK] inhibitor, 5 nM, Selleckchem, Houston, 24 h).

Lentiviral over-expression and silencing vectors of miR-331-3p and SCARA5 lentiviral over-expression vector (the 5’-untranslated region [UTR] and 3’-UTR were added at the front and end of the CDS region of SCARA5 [433–1920 region of NM_173833.6] to form the Lv-SCARA5 vector) were acquired from Genechem, and cloned into inactivated lentiviral vector (pSicoR). In addition, the synthesized oligonucleotides were annealed and introduced restriction sites of *Hpa 1 and Xho I*, followed by restriction digestion and DNA sequencing to identify positive clones. Next, 293 T cells (CRL-1573, ATCC) were transfected with vectors encompassing positive intersections and packaging vectors, psPAX2 and pMD2. The supernatant encompassing lentivirus was yielded every 12 h during 24 h to 72 h after transduction, and then filtered using 0.45 μm cellulose acetate filters to obtain the final virus titer 10^9^ TU/mL. A day prior to transduction, log-phase CAFs, SW1990, and PANC-1 cells were incubated in a 6-well plate at 1 × 10^5^ cells/mL. Once cells achieved 50–70% confluence, the medium was removed and a 1 mL of 1: 10 dilution of viral medium was added along with Polybrene (H8761, Solarbio, Beijing, China) to culture the cells in a humidified incubator with 5% CO_2_ at 37°C. Subsequent to 24-h transduction, cells were selected with a final concentration of 600 mg/L G418 that was renewed every 3 days. Following 12 days, non-transfected CAFs, SW1990, and PANC-1 cells were dead, and 48% transfected cells were still alive, which were cultured further in the medium with 300 mg/L G418 to obtain stable transfected cells.

### EV isolation

Exponentially growing CAFs were incubated in a 6-well plate with DMEM encompassing 10% EV-free serum in a humidified incubator with 5% CO_2_ at 37°C. Subsequent to 2 days, cell culture supernatant was attained and filtered through a 0.22 μm filter to discard cell clumps. Next, the filtered medium underwent 10-min centrifugation at 500 g and 4°C to discard cells, 10-min centrifugation at 12000 g to discard dead cells, 30-min ultracentrifugation at 100000 g to discard cell debris, and then 40-min ultracentrifugation at 100000 g to discard the supernatant. The harvested pellet was resuspended with sterile PBS before 90-min ultracentrifugation at 100000 g and 4°C. Next, the pellet was resuspended with 10 mL PBS and received 90-min ultracentrifugation again at 100000 g and 4°C. The supernatant was removed, and the pellet was regarded as the EVs that were resuspended with 100 μL PBS and preserved at −80°C for subsequent experimentation.

### Transmission electron microscopy (TEM)

The EVs were pelleted and immediately immobilized in 2.5% glutaraldehyde at 4°C before gradient alcohol dehydration and epoxy resin embedding. Next, the EV sections received uranyl acetate and lead citrate staining, followed by observation using TEM (JEM-1010, JEOL, Tokyo, Japan).

### Nanoparticle tracking analysis (NTA)

EVs were diluted using PBS to a concentration of 10^6^ cells/mL – 10^9^ cells/mL. Next, samples were loaded and analyzed with a nanosight analyzer (Nanosight NS300, Malvern, UK). The experiment was repeated 3 times.

### EV internalization by SW1990 and PANC-1 cells

EVs were incubated with 2 μM red fluorescent dye PKH67 (Sigma-Aldrich) for 10 min following the manufacturer’s manuals before 90-min centrifugation at 120000 g to discard the unincorporated dye. Afterward, 6-h co-incubation of EVs with SW1990 and PANC-1 cells was implemented at 37°C before 15-min cell immobilization using 4% paraformaldehyde. Subsequent to cell staining with 4’-6-diamidino-2-phenylindole (DAPI; 0.5 µg/mL; Invitrogen, Carlsbad, CA), cell observation was conducted under a confocal laser microscope (FV-1000, Olympus, Japan).

### Cell counting kit-8 (CCK-8) assay

CCK-8 kits (WH5199, Biotechwell, Shanghai, China) were adopted to detect cell proliferation. Logarithmically growing cells were diluted in DMEM encompassing 10% FBS to 5 × 10^4^ cells/mL. Next, 100 μL diluted cells were seeded in a 96-well plate before the supplementation of 10 μL CCK-8 at variable time points. Following 2-h cell incubation at 37°C, the absorbance (A) was assessed using a Multiskan FC microplate reader (51119080, Thermo Fisher Scientific) at 450 nm. Mean value was calculated from five parallel measurements.

### Transwell assay

With respect to cell migration, 200 μL SW1990 and PANC-1 cells cultured in the serum-free medium were seeded in the upper layer at 5 × 10^4^, while the bottom layer was added with 800 μL medium with 20% FBS, followed by a 16-h culture in a humidified incubator at 37°C. Transwell chamber was removed, followed by formaldehyde rinsing for 10 min. Subsequent to 30-min cell staining with 0.1% crystal violet at ambient temperature, cells on the surface were discarded using a cotton ball. Finally, cells underwent observation, photographing, and counting under an inverted microscope.

For cell invasion assay, prior to cell addition, Matrigel (356235, BD-Biocoat, Franklin Lakes, NJ) stored at −80°C was balanced overnight at 4°C to melt to the liquid state. Matrigel was supplemented to chambers (50 μL/well). Following solidification at 37°C, cell suspension was supplemented to chambers before 24-h culture and 30-min 0.1% crystal violet staining. Cells on the upper surface were discarded using cotton balls. A total of 6 random fields underwent microscopic observation and photographing to calculate the number of migration/invasion cells.

### Dual-luciferase reporter assay

The synthesized SCARA5 3’-UTR was introduced a pmirGLO vector (Promega, Madison, WI). The mutation was designed using SCARA5 wild type (WT) sequence, which was digested with a restriction enzyme and ligated with T4 DNA ligase into the pmirGLO vector. The WT and mutant type (MUT) were co-transfected with miR-331-3p mimic or miR-331-3p inhibitor into 293 T cells. Subsequent to 48-h transfection, the cells were lysed and subjected to a Dual-Luciferase Reporter assay (Promega). The luciferase activity was evaluated under Luminometer TD-20/20 (E5311, Promega) and calculated as the ratio of firefly luciferase activity to renilla luciferase activity, which was normalized to the ratio in the NC-mimic group. The experiment was repeated three times independently to obtain the mean value.

### Co-culture of Cy3 fluorescently labeled CAFs with PC cells

Cy3-labeled miR-331-3p (miR-331-3p-Cy3; GenePharma) was transduced into CAFs using the Lipofectamine 2000 reagent (11668019, Invitrogen) to identify the transfer of EVs-miR-331-3p. CAFs expressing miR-331-3p-Cy3 were seeded in a 6-well plate and co-cultured with PC cells in a Transwell chamber (3412, Corning Incorporated, Corning, NY) for 2–4 days. Following 10-min PC cell immobilization using 4% paraformaldehyde, cells underwent 5-min permeabilizing with PBS encompassing 0.5% Triton X-100 and 5-min DAPI (C1002, Beyotime) staining for nuclear staining. Finally, a confocal microscope was employed to observe PC cells.

### miRNA and mRNA quantitative analyses

miRNA analysis was carried out as follows: Total RNA Purification Micro kits (35350, Norgen Biotek, Canada) were adopted applied to isolate the RNA content from EVs, and 20 μL elution solution E was used to recover RNA that was stored at −80°C. A NanoDrop ND-2000 spectrophotometer was utilized to quantify the concentration and amount of the extracted RNA. For mRNA detection, cDNA was generated using Reverse Transcription kits (RR047A, Takara, Japan). For miRNA detection, Poly(A) Tailing kits (B532451, Sangon; encompassing universal PCR primer R and U6 universal PCR primer R) were adopted for reverse transcription to generate cDNA of miRNA with PolyA tail. Reverse transcription quantitative polymerase chain reaction (RT-qPCR) was implemented following the manuals of miScript SYBR® Green PCR kits (218073, Qiagen GmbH, Hilden, Germany).

mRNA analysis was carried out as follows: Total RNA content was isolated with the TRIzol reagent (15596026, Invitrogen) before generation of cDNA from 2 μg RNA with TaqMan reverse transcription reagents (4304134, Invitrogen) according to the procedures. Next, qPCR was conducted on StepOnePlus™ Real-Time PCR System (4376600, Applied Biosystems, Thermo Fisher Scientific).

Primers are manifested in Supplementary Table 1. Spiked-in cel-miR-39 (NGB-59000, Norgen Biotek, Canada) and U6 were employed as the normalizer for miR-331-3p, while β-actin was used as the normalizer for other mRNAs. SYBR® Premix Ex Taq™ (Perfect Real Time) (DRR041S, Takara, Japan) was applied to perform qPCR, and the 2^−ΔΔCt^ method was applied to calculate the relative gene expression.

### Enzyme-linked immunosorbent assay (ELISA)

After 48 h of treatment, the supernatant of SW1990 and PANC-1 cells was harvested and prepared for ELISA detection. The cell supernatant received 15-min centrifugation at 4°C, after which matrix metalloproteinase-2 (MMP-2) and matrix metalloproteinase-9 (MMP-9) concentration was identified in light of the manuals of the ELISA kits (R&D Systems, Minneapolis, MN).

### Western blot analysis

Following 48-h treatment, SW1990 and PANC-1 cells were harvested, and protein samples were prepared for Western blot analysis. Cells or tissues were incubated with 1 mL of cell lysis buffer (encompassing protease inhibitor) (P0013 J, Beyotime Biotechnology, Beijing, China) for 45 min at 4°C with shaking every 10 min before 30-min centrifugation at 4000 g. Next, the supernatant was acquired and subjected to bicinchoninic acid kit (PC0020, Solarbio, Beijing, China) to quantify protein concentration. Following separation using sodium dodecyl sulfate-polyacrylamide gel electrophoresis, 50 μg of proteins were electroblotted onto a polyvinylidene fluoride membrane (66485, PALL, Port Washington, NY). The membrane underwent 2-h 5% skim milk blocking at ambient temperature before overnight probing at 4°C with primary antibodies from Cell Signaling Technologies (CST, Danvers, MA) against FAK (#3285, 1: 1000), MMP-9 (#3852, 1: 1000), and β-actin (#4967, 1: 1000, CST), antibodies from Invitrogen to phosphorylated (p)-FAK (44–624 G, 1: 1000), and antibodies from Abcam (Cambridge, UK) to MMP-2 (ab92536, 1: 1000), CD63 (ab134045, 1: 1000), CD81 (ab109201, 1: 1000), Alix (ab225555, 1: 2000), golgi matrix protein 130 (GM130, #12480, 1: 1000), alpha-smooth muscle actin (α-SMA; ab32575, 1: 1000), and fibroblast activation protein (FAP, ab53066, 1: 1000), followed by re-probing with horseradish peroxidase-labeled secondary antibody IgG (ab97051, 1: 1000, Abcam) for 1 h at ambient temperature. The membrane was then incubated with enhanced chemiluminescence (BM101, Biomiga, San Diego, CA) and developed under conditions void of light. β-actin served as the loading control.

### Immunohistochemistry

Tumor tissue samples were immobilized with 10% neutral formalin solution before paraffin-embedding and sectioning with an ultramicrotome. Sectioned samples were deparaffinized with xylene before graded alcohol rehydration. The endogenous peroxidase activity was blocked through incubation with 3% hydrogen peroxide. The slides were then heated in 10 mM sodium citrate (pH 6.0) for 30 min, blocked with 10% normal goat serum for 15 min, and immunostained with primary antibodies against Ki67 (ab15580, 1:1000, Abcam) and p-FAK (44–624 G, 1:200, Invitrogen) overnight in a wet room at 4°C. The following day, the slides were re-probed with a secondary antibody for 1 h at ambient temperature before 3,3’-diaminobenzidine tetrahydrochloride developing. Each group of samples contained at least three tissues, and three fields were randomly selected from each tissue sample and photographed under a microscope, with the proportion of positive cells counted.

### Tissue in situ hybridization

Five-μm-thick tissue sections were dewaxed with xylene and dehydrated with an ascending series of alcohols. The sections were incubated with a hot plate in a citrate buffer (10 nmol/L, pH 6) at 100–103°C for 15 min and immediately heated with 10 μg/mL Protease (Sigma-Aldrich) in a HybEZ hybridization oven (Advanced Cell Diagnostics, Hayward, CA) at 40°C for 30 min. Afterward, the sections were incubated with fluorescein isothiocyanate-miR-331-3p probe for hybridization and development.

### Tumor xenograft in nude mice

BALB/c female nude mice (aged 8 − 12 weeks) were purchased from the Institute of Zoology, Chinese Academy of Sciences. The procured mice were fed with fine-grained food, allowing free access of water, natural light in the feeding room with a 12 h light/dark cycle every day. Subsequently, 30 nude mice were randomly selected and each mouse was subcutaneously inoculated with the cell suspension (PANC-1 cells suspended with PBS to concentration of 5 × 10^7^ cells/0.1 mL), and the remaining 15 mice were used as control. The maximum tumor diameter (L) and minimum diameter (W) were measured with a digital caliper every 3 days, and the tumor volume (V) was calculated according to the following formula: V = W^2^ × L × 0.52. When the tumor reached to 50 mm^3^, the 30 inoculated mice were randomly grouped into 2 groups, 15 mice of each, which were injected intravenously with EVs-NC (25 μg of CAFs-derived EVs with lentiviral empty vector control) and EVs-miR-331-3p (25 μg of CAFs-derived EVs with lentiviral miR-331-3p over-expression construct), respectively. The injection was repeated every 3 d and continued for 3 weeks. The tumor volume was measured and calculated for every 3 d as well. When nude mice lost more than 20% body weight or tumor volume exceeded 1200 mm^3^, the nude mice were euthanized and tumors were removed and weighed.

### Statistical analysis

Statistical analyses were performed using the SPSS 21.0 statistical software (IBM Corp. Armonk, NY). Measurement data were presented as mean ± standard deviation. Data from the two groups were analyzed using unpaired *t* test, while data among multiple groups were analyzed *via* one-way analysis of variance (ANOVA) and Tukey’s post-hoc test, and data among multiple groups at different time points were analyzed using repeated measures ANOVA and Bonferroni post-hoc test. The Kaplan–Meier method was adopted to analyze the survival rate to evaluate the relationship between prognosis and miR-331-3p. Pearson’s correlation coefficient was conducted to analyze the correlation between miR-331-3p and SCARA5 expression. A value of *p < .05* was regarded statistically significant.

## Supplementary Material

Supplemental Material
