# Supplementary material for: Carcinoma-associated fibroblasts release microRNA-331-3p containing extracellular vesicles to exacerbate the development of pancreatic cancer via the SCARA5-FAK axis
Source: Cancer Biol Ther. 2022 May 5;23(1):378–92. doi: 10.1080/15384047.2022.2041961 (PMC9090287; doi:10.1080/15384047.2022.2041961)
Supplement: Supplemental Material [file KCBT_A_2041961_SM6447.zip › supplementary/Supplementary Table 1 (1).docx]

**Supplementary Table 1.** Primer list for RT-qPCR

| Target genes | Primer sequences |
| --- | --- |
| miR-331-3p | F: 5’-AGTTTGGTTTTGTTTGGGTTTGT-3’ |
|  | R: 5’-TGGTTCTAGGATAGGCCCAG-3’ |
| U6 | F: 5’-AAAGCAAATCATCGGACGACC-3’ |
|  | R: 5’-GTACAACACATTGTTTCCTCGGA-3’ |
| SCARA5 | F: 5’-TCTTCATCTTAGCAGTGTCCAG-3’ |
|  | R: 5’-GCAACGAGTCTGACTGGTTC-3’ |
| α-SMA | F: 5’-CCTTGAGAAGAGTTACGAGTTGC-3’ |
|  | R: 5’-ATGATGCTGTTGTAGGTGGTTT-3’ |
| FAP | F: 5’-TGTGCATTGTCTTACGCCCT-3’ |
|  | R: 5’-CCGATCAGGTGATAAGCCGT-3’ |
| β-actin | F: 5’-TCCTGTGGCATCCACGAAACTACA-3’ |
|  | R: 5’-ACCAGACAGCACTGTGTTGGCATA-3’ |
