# Supplementary material for: Carcinoma-associated fibroblasts release microRNA-331-3p containing extracellular vesicles to exacerbate the development of pancreatic cancer via the SCARA5-FAK axis
Source: Cancer Biol Ther. 2022 May 5;23(1):378–92. doi: 10.1080/15384047.2022.2041961 (PMC9090287; doi:10.1080/15384047.2022.2041961)
Supplement: Supplemental Material [file KCBT_A_2041961_SM6447.zip › supplementary/Supplementary_Figure_Legends.docx]

**Supplementary Figure Legends**

**Supplementary Figure 1. Effect of NFs-derived EVs on the proliferation, migration and invasion of SW1990 and PANC-1 cells.**

A. TEM visualization of the morphology of NFs-derived EVs. B. Size distribution of the NFs-derived EVs assessed by NTA. C. Expression of EVs-related proteins determined by western blot analysis. D. Proliferation of SW1990 and PANC-1 cells after co-culture with NFs-derived EVs for 48 h measured by CCK-8 assay. E. Migration and invasion of SW1990 and PANC-1 cells after co-culture with NFs-derived EVs for 48 h measured by Transwell assay. F. Expression of MMP-2 and MMP-9 in SW1990 and PANC-1 cells after co-culture with NFs-derived EVs for 48 h determined by western blot analysis. Experiments were repeated 3 times. ** indicates *p* < 0.01, *** indicates *p* < 0.001, and **** indicates *p* < 0.0001.

**Supplementary Figure 2. Representative western blots for the densitometric quantification of panel 1K (A), 2F (B), 5C (C), and 6G (D).**

**Supplementary Figure 3. miR-331-3p is highly expressed in CAFs.**

Expression of miR-331-3p in NFs, CAFs and PC cell lines determined by RT-qPCR. Experiments were repeated 3 times. ** indicates *p* < 0.01, and *** indicates *p* < 0.001.
